# BaCo_2_(AsO_4_)_2_

**DOI:** 10.1107/S1600536808025865

**Published:** 2008-08-16

**Authors:** Tamara Đordević

**Affiliations:** a Institut für Mineralogie und Kristallographie, Universität Wien-Geozentrum, Althanstrasse 14, A-1090 Vienna, Austria

## Abstract

Suitable single crystals of the title compound, barium dicobalt(II) bis­[orthoarsenate(V)], were prepared under hydro­thermal conditions. This phase belongs to a series of compounds with general formula *AM*_2_(*X*O_4_)_2_, where *A* = alkaline earth metal, *M* = Mg or a divalent first-row transition element, and *X* = P, As or V. BaCo_2_(AsO_4_)_2_ is isotypic with BaNi_2_(*X*O_4_)_2_ (*X* = P, V or As) and is characterized by brucite-like sheets of edge-sharing CoO_6_ octa­hedra (3 symmetry) parallel to (001), with one-third of the octa­hedral positions being vacant. The sheets are capped above and below by AsO_4_ tetra­hedra (3 symmetry) and are inter­connected by distorted BaO_12_ cubocta­hedra (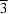 symmetry).

## Related literature

For isostructural compounds, see: Eymond *et al.* (1969*a* ▶,*b* ▶); Bircsak & Harrison (1998 ▶); El-Bali *et al.* (1999 ▶); Faza *et al.* (2001 ▶); Rogado *et al.* (2002 ▶); Wichmann, & Müller-Buschbaum (1984 ▶). For magnetic properties of BaCo_2_(AsO_4_)_2_, see: Dojčilović *et al.* (1994 ▶); Regnault *et al.* (2006 ▶). For related compounds, see: Effenberger & Pertlik (1993 ▶); El-Bali *et al.* (1993*a* ▶,*b* ▶); Hemon & Courbion (1990 ▶); Kreidler & Hummel (1961 ▶); Lucas *et al.* (1998 ▶); Mihajlović *et al.* (2004 ▶); Moquine *et al.* (1993 ▶); Osterloh & Müller-Buschbaum (1994 ▶). For general background, see: Brese & O’Keeffe (1991 ▶).

## Experimental

### 

#### Crystal data

BaCo_2_(AsO_4_)_2_
*M* *_r_* = 533.04
Hexagonal, 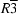

*a* = 5.007 (1) Å
*c* = 23.491 (5) Å
*V* = 510.02 (18) Å^3^
*Z* = 3
Mo *K*α radiation
μ = 20.22 mm^−1^
*T* = 293 (2) K
0.09 × 0.05 × 0.05 mm

#### Data collection

Nonius KappaCCD diffractometer
Absorption correction: multi-scan (Otwinowski & Minor, 1997 ▶; Otwinowski *et al.*, 2003 ▶) *T*_min_ = 0.221, *T*_max_ = 0.362
681 measured reflections
341 independent reflections
336 reflections with *I* > 2σ(*I*)
*R*_int_ = 0.009

#### Refinement

*R*[*F* ^2^ > 2σ(*F* ^2^)] = 0.014
*wR*(*F* ^2^) = 0.038
*S* = 1.30
341 reflections
22 parameters
Δρ_max_ = 0.96 e Å^−3^
Δρ_min_ = −1.09 e Å^−3^

### 

Data collection: *COLLECT* (Nonius, 2002 ▶); cell refinement: *SCALEPACK* (Otwinowski & Minor, 1997 ▶); data reduction: *DENZO-SMN* (Otwinowski *et al.*, 2003 ▶); method used to solve structure: starting parameters from an isostructural compound (Eymond *et al.*, 1969*a* ▶); program(s) used to refine structure: *SHELXL97* (Sheldrick, 2008 ▶) and *WinGX* (Farrugia, 1999 ▶); molecular graphics: *ATOMS* (Dowty, 2000 ▶); software used to prepare material for publication: *publCIF* (Westrip, 2008 ▶).

## Supplementary Material

Crystal structure: contains datablocks global, I. DOI: 10.1107/S1600536808025865/wm2189sup1.cif

Structure factors: contains datablocks I. DOI: 10.1107/S1600536808025865/wm2189Isup2.hkl

Additional supplementary materials:  crystallographic information; 3D view; checkCIF report

## Figures and Tables

**Table 1:** Selected bond lengths (Å)

Ba1—O1	2.9344 (8)
Ba1—O2	3.1611 (17)
Co1—O2^i^	2.0791 (17)
Co1—O2	2.1017 (17)
As1—O1	1.656 (3)
As1—O2	1.7050 (17)

Symmetry code: (i) 
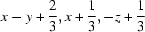
.

**Fig. 1.**
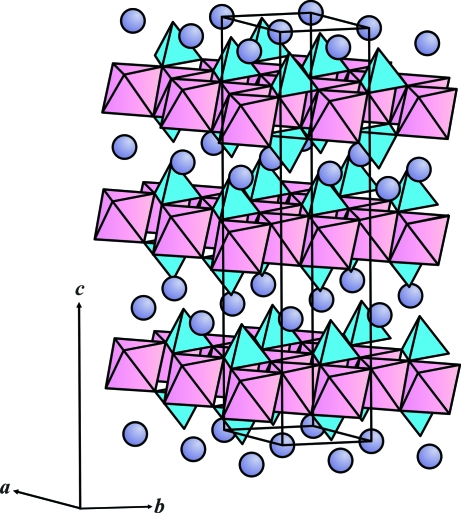
The crystal structure of BaCo2(AsO4)2 with sheets consisting of CoO6 octahedra and AsO4 tetrahedra (both in polyhedral representation) parallel to (001). Ba atoms are displayed as spheres.

**Fig. 2.**
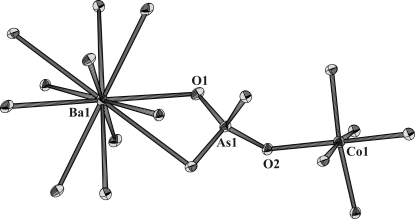
Coordination of Ba and Co with atoms displayed as ellipsoids at the 50% probability level.

## supplementary crystallographic information

### Comment

The crystal structures of phosphates, arsenates and vanadates with the general formula *A**M*_2_(*X*O_4_)_2_ where *A* = alkaline earth metal, *M* = Mg or divalent first row transition elements and *X* = P, As or V, are relatively well known (Bircsak & Harrison, 1998; El-Bali *et al.*, 1993*a*,*b*, 1999; Eymond *et al.*, 1969*a*,*b*; Hemon & Courbion, 1990; Kreidler & Hummel, 1961; Lucas *et al.*, 1998; Moquine *et al.*, 1993; Osterloh & Müller-Buschbaum, 1994; Wichmann & Müller-Buschbaum, 1984, and references therein). These phases adopt different structure types and exhibit interesting physical properties (phase transitions, magnetism). Six compounds, *viz.* BaMg_2_(AsO_4_)_2_, BaCo_2_(AsO_4_)_2_ (Eymond *et al.*, 1969*b*), BaNi_2_(AsO_4_)_2_ (Eymond *et al.*, 1969*a*,*b*), BaCo_2_(PO_4_)_2_ (Bircsak & Harrison, 1998), BaNi_2_(PO_4_)_2_ (El-Bali *et al.*, 1999; Faza *et al.*, 2001) and BaNi_2_(VO_4_)_2_ (Wichmann & Müller-Buschbaum, 1984; Rogado *et al.*, 2002) are isostructural with the title compound, BaCo_2_(AsO_4_)_2_. The underlying crystal structure was described for the first time for BaNi_2_(AsO_4_)_2_ by Eymond *et al.* (1969*a*). Although two-dimensional magnetic properties of these compounds have been widely studied (Dojčilović *et al.*, 1994; Regnault *et al.*, 2006, and references therein), a full determination of their crystal structures was not given for all members of this structural family. Hydrothermal synthesis and the crystal structure of  BaCo_2_(AsO_4_)_2_ are presented in this communication.

Besides BaCoAs_2_O_7_ (Mihajlović *et al.*, 2004), BaCo_2_(AsO_4_)_2_ represents the second compound structurally characterised in the system BaO–CoO–As_2_O_5_. Its crystal structure is made up of brucite-like sheets of edge-sharing CoO_6_ octahedra parallel to (001), with one-thirds of the octahedral positions being vacant. AsO_4_ tetrahedra are situated above and below the sheets. The resulting anionic [Co_2_(AsO_4_)_2_]^2-^ layers are stacked with a sequence *ABCABC* along [001] and are laterally displaced by Δ*x* = 2/3*a*, with Δ*x* = 1/3*b* between the layers. Adjacent layers are interconnected by BaO_12_ polyhedra to form a three-dimensional framework (Fig. 1).

The Ba1 atom has site symmetry 3 and is coordinated by twelve oxygen atoms (Fig. 2) with an average Ba1—O bond length of 3.048 Å. This bond length compares well with the average bond length for Ba—O distances of 3.015 Å in the isostructural Ni compound BaNi_2_(AsO_4_)_2_ (Eymond *et al.*, 1969*a*,*b*). The Ba1 atom is bonded to six O1 and six O2 atoms, resulting in a distorted cuboctahedral coordination polyhedron. The Co1 atom has site-symmetry 3 and is octahedrally coordinated to O1 atoms with a mean Co1—O1 distance of 2.091 Å. The As1O_4_ tetrahedron (3 symmetry) exhibits an average bond length of 1.692 Å, with two symmetrically independent As1—O bonds of 1.656 (3) Å (O1) and of 1.7050 (17) Å (O2, 3×) due to the different other coordination partners of the two oxygen atoms. O1, which bridges the barium ions, shows a shorter As1—O bond than O2, which bridges the cobalt ions (Fig. 2).

Bond-valence calculations for all atoms, using the parameters of Brese & O'Keeffe (1991), give 1.63 v.u. (valence units) for Ba1, 2.04 v.u. for Co1, 4.89 v.u. for As1, and 1.53 v.u and 1.96 v.u for O1 and O2, respectively. If one takes into account that the O2 atom is bonded to two Co1, one As1, and one Ba1 atom, and the O1 atom is bonded to one As1 and three Ba1 neighbours, the calculated values are close to the theoretical valences. However, it is worth mentioning that in BaCo_2_(AsO_4_)_2_ and all isostructural compounds the Ba1 atom is strongly undersaturated in terms of its bond valence.

### Experimental

Single crystals of BaCo_2_(AsO_4_)_2_ were obtained as reaction products from mixtures of Ba(OH)_2_.8H_2_O (Merck, >97%), Co(OH)_2_ (Alfa Products) and As_2_O_5_ (Alfa Products, >99.9%) under hydrothermal conditions. The mixture was transferred into a Teflon vessel and filled to approximately 70% of its inner volume with distilled water (pH of the resulting solution was ≈ 2.5). Finally, the vessel was enclosed into a stainless steel autoclave and was heated from room temperature to 493 K (2 h), held at 493 K for 24 h, then cooled to 393 K within 14 h, kept at this temperature for 24 h, and finally cooled to room temperature within 4 h. At the end of the reaction the pH was ≈ 6. The solid reaction products were filtered and washed thoroughly with distilled water. BaCo_2_(AsO_4_)_2_ (yield *ca* 50%) crystallized as transparent pink crystals and was accompanied with prismatic blue–green crystals of BaCoAs_2_O_7_ (Mihajlović *et al.*, 2004) (yield *ca* 30%) and Co_2_As_2_O_7_(H_2_O)_2_ (Effenberger & Pertlik, 1993) (yield *ca* 15%), besides very few prismatic blue crystals of an yet unidentified compound (yield *ca* 5%). All crystals were up to 0.2 mm in length.

### Refinement

The crystal structure was refined with the atomic coordinates of BaNi_2_(AsO_4_)_2_ (Eymond *et al.*, 1969*a*) as starting parameters in the hexagonal setting of space group *R*3.

### Crystal data

**Table:**

BaCo_2_(AsO_4_)_2_	*Z* = 3
*M**_r_* = 533.04	*F*_000_ = 720
Hexagonal, *R*3	*D*_x_ = 5.206 Mg m^−^^3^
Hall symbol: -R 3	Mo *K*α radiation λ = 0.71073 Å
*a* = 5.007 (1) Å	Cell parameters from 675 reflections
*b* = 5.007 (1) Å	θ = 1.0–30.0º
*c* = 23.491 (5) Å	µ = 20.22 mm^−^^1^
α = 90º	*T* = 293 (2) K
β = 90º	Pseudo-hexagonal plate, pink
γ = 120º	0.09 × 0.05 × 0.05 mm
*V* = 510.02 (18) Å^3^	

### Data collection

**Table:**

Nonius KappaCCD diffractometer	341 independent reflections
Radiation source: fine-focus sealed tube	336 reflections with *I* > 2σ(*I*)
Monochromator: graphite	*R*_int_ = 0.009
*T* = 293(2) K	θ_max_ = 30.0º
φ and ω scans	θ_min_ = 2.6º
Absorption correction: multi-scan(Otwinowski & Minor, 1997; Otwinowski *et al.*, 2003)	*h* = −7→7
*T*_min_ = 0.221, *T*_max_ = 0.362	*k* = −5→5
681 measured reflections	*l* = −33→33

### Refinement

**Table:**

Refinement on *F*^2^	Primary atom site location: isomorphous structure methods
Least-squares matrix: full	*w* = 1/[σ^2^(*F*_o_^2^) + (0.0133*P*)^2^ + 3.104*P*] where *P* = (*F*_o_^2^ + 2*F*_c_^2^)/3
*R*[*F*^2^ > 2σ(*F*^2^)] = 0.014	(Δ/σ)_max_ < 0.001
*wR*(*F*^2^) = 0.038	Δρ_max_ = 0.96 e Å^−^^3^
*S* = 1.30	Δρ_min_ = −1.09 e Å^−^^3^
341 reflections	Extinction correction: SHELXL97 (Sheldrick, 2008), Fc^*^=kFc[1+0.001xFc^2^λ^3^/sin(2θ)]^-1/4^
22 parameters	Extinction coefficient: 0.0118 (5)

### Special details

**Table:**

Geometry. All esds (except the esd in the dihedral angle between two l.s. planes) are estimated using the full covariance matrix. The cell esds are taken into account individually in the estimation of esds in distances, angles and torsion angles; correlations between esds in cell parameters are only used when they are defined by crystal symmetry. An approximate (isotropic) treatment of cell esds is used for estimating esds involving l.s. planes.
Refinement. Refinement of F^2^ against ALL reflections. The weighted R-factor wR and goodness of fit S are based on F^2^, conventional R-factors R are based on F, with F set to zero for negative F^2^. The threshold expression of F^2^ > 2sigma(F^2^) is used only for calculating R-factors(gt) etc. and is not relevant to the choice of reflections for refinement. R-factors based on F^2^ are statistically about twice as large as those based on F, and R- factors based on ALL data will be even larger.

### Fractional atomic coordinates and isotropic or equivalent isotropic displacement parameters (Å^2^)

**Table:**

	*x*	*y*	*z*	*U*_iso_*/*U*_eq_	
Ba1	0.0000	0.0000	0.0000	0.01170 (15)	
Co1	0.0000	0.0000	0.17014 (2)	0.00614 (16)	
As1	0.3333	0.6667	0.091941 (18)	0.00473 (15)	
O1	0.3333	0.6667	0.02145 (13)	0.0118 (6)	
O2	0.0163 (4)	0.3375 (4)	0.11476 (7)	0.0077 (3)	

### Atomic displacement parameters (Å^2^)

**Table:**

	*U*^11^	*U*^22^	*U*^33^	*U*^12^	*U*^13^	*U*^23^
Ba1	0.01138 (17)	0.01138 (17)	0.0124 (2)	0.00569 (8)	0.000	0.000
Co1	0.00474 (19)	0.00474 (19)	0.0089 (3)	0.00237 (9)	0.000	0.000
As1	0.00414 (16)	0.00414 (16)	0.0059 (2)	0.00207 (8)	0.000	0.000
O1	0.0149 (9)	0.0149 (9)	0.0055 (13)	0.0075 (5)	0.000	0.000
O2	0.0055 (7)	0.0050 (7)	0.0116 (8)	0.0019 (6)	0.0017 (6)	0.0020 (6)

### Geometric parameters (Å, °)

**Table:**

Ba1—O1^i^	2.9344 (8)	Ba1—O2^ix^	3.1611 (17)
Ba1—O1^ii^	2.9344 (8)	Co1—O2^x^	2.0791 (17)
Ba1—O1^iii^	2.9344 (8)	Co1—O2^xi^	2.0791 (17)
Ba1—O1	2.9344 (8)	Co1—O2^xii^	2.0792 (17)
Ba1—O1^iv^	2.9344 (8)	Co1—O2^vii^	2.1017 (17)
Ba1—O1^v^	2.9344 (8)	Co1—O2	2.1017 (17)
Ba1—O2^vi^	3.1611 (17)	Co1—O2^vi^	2.1017 (17)
Ba1—O2	3.1611 (17)	As1—O1	1.656 (3)
Ba1—O2^vii^	3.1611 (17)	As1—O2^xiii^	1.7050 (17)
Ba1—O2^iii^	3.1611 (17)	As1—O2	1.7050 (17)
Ba1—O2^viii^	3.1611 (17)	As1—O2^xiv^	1.7050 (17)
			
O1^i^—Ba1—O1^ii^	180.00 (12)	O2^vii^—Ba1—O2^iii^	126.22 (5)
O1^i^—Ba1—O1^iii^	117.11 (3)	O1^i^—Ba1—O2^viii^	52.94 (6)
O1^ii^—Ba1—O1^iii^	62.89 (3)	O1^ii^—Ba1—O2^viii^	127.06 (6)
O1^i^—Ba1—O1	62.89 (3)	O1^iii^—Ba1—O2^viii^	82.85 (6)
O1^ii^—Ba1—O1	117.11 (3)	O1—Ba1—O2^viii^	97.15 (6)
O1^iii^—Ba1—O1	180.00 (12)	O1^iv^—Ba1—O2^viii^	106.72 (6)
O1^i^—Ba1—O1^iv^	117.11 (3)	O1^v^—Ba1—O2^viii^	73.28 (6)
O1^ii^—Ba1—O1^iv^	62.89 (3)	O2^vi^—Ba1—O2^viii^	180.00 (7)
O1^iii^—Ba1—O1^iv^	117.11 (3)	O2—Ba1—O2^viii^	126.22 (5)
O1—Ba1—O1^iv^	62.89 (3)	O2^vii^—Ba1—O2^viii^	126.22 (5)
O1^i^—Ba1—O1^v^	62.89 (3)	O2^iii^—Ba1—O2^viii^	53.78 (5)
O1^ii^—Ba1—O1^v^	117.11 (3)	O1^i^—Ba1—O2^ix^	82.85 (6)
O1^iii^—Ba1—O1^v^	62.89 (3)	O1^ii^—Ba1—O2^ix^	97.15 (6)
O1—Ba1—O1^v^	117.11 (3)	O1^iii^—Ba1—O2^ix^	106.72 (6)
O1^iv^—Ba1—O1^v^	180.00 (12)	O1—Ba1—O2^ix^	73.28 (6)
O1^i^—Ba1—O2^vi^	127.06 (6)	O1^iv^—Ba1—O2^ix^	52.94 (6)
O1^ii^—Ba1—O2^vi^	52.94 (6)	O1^v^—Ba1—O2^ix^	127.06 (6)
O1^iii^—Ba1—O2^vi^	97.15 (6)	O2^vi^—Ba1—O2^ix^	126.22 (5)
O1—Ba1—O2^vi^	82.85 (6)	O2—Ba1—O2^ix^	126.22 (5)
O1^iv^—Ba1—O2^vi^	73.28 (6)	O2^vii^—Ba1—O2^ix^	180.00 (3)
O1^v^—Ba1—O2^vi^	106.72 (6)	O2^iii^—Ba1—O2^ix^	53.78 (5)
O1^i^—Ba1—O2	73.28 (6)	O2^viii^—Ba1—O2^ix^	53.78 (5)
O1^ii^—Ba1—O2	106.72 (6)	O2^x^—Co1—O2^xi^	92.91 (7)
O1^iii^—Ba1—O2	127.06 (6)	O2^x^—Co1—O2^xii^	92.91 (7)
O1—Ba1—O2	52.94 (6)	O2^xi^—Co1—O2^xii^	92.91 (7)
O1^iv^—Ba1—O2	97.15 (6)	O2^x^—Co1—O2^vii^	92.34 (6)
O1^v^—Ba1—O2	82.85 (6)	O2^xi^—Co1—O2^vii^	174.36 (6)
O2^vi^—Ba1—O2	53.78 (5)	O2^xii^—Co1—O2^vii^	88.86 (9)
O1^i^—Ba1—O2^vii^	97.15 (6)	O2^x^—Co1—O2	174.36 (6)
O1^ii^—Ba1—O2^vii^	82.85 (6)	O2^xi^—Co1—O2	88.86 (9)
O1^iii^—Ba1—O2^vii^	73.28 (6)	O2^xii^—Co1—O2	92.34 (6)
O1—Ba1—O2^vii^	106.72 (6)	O2^vii^—Co1—O2	85.72 (7)
O1^iv^—Ba1—O2^vii^	127.06 (6)	O2^x^—Co1—O2^vi^	88.86 (9)
O1^v^—Ba1—O2^vii^	52.94 (6)	O2^xi^—Co1—O2^vi^	92.34 (6)
O2^vi^—Ba1—O2^vii^	53.78 (5)	O2^xii^—Co1—O2^vi^	174.36 (6)
O2—Ba1—O2^vii^	53.78 (5)	O2^vii^—Co1—O2^vi^	85.72 (7)
O1^i^—Ba1—O2^iii^	106.72 (6)	O2—Co1—O2^vi^	85.72 (7)
O1^ii^—Ba1—O2^iii^	73.28 (6)	O1—As1—O2^xiii^	108.33 (6)
O1^iii^—Ba1—O2^iii^	52.94 (6)	O1—As1—O2	108.33 (6)
O1—Ba1—O2^iii^	127.06 (6)	O2^xiii^—As1—O2	110.59 (5)
O1^iv^—Ba1—O2^iii^	82.85 (6)	O1—As1—O2^xiv^	108.33 (6)
O1^v^—Ba1—O2^iii^	97.15 (6)	O2^xiii^—As1—O2^xiv^	110.59 (5)
O2^vi^—Ba1—O2^iii^	126.22 (5)	O2—As1—O2^xiv^	110.59 (5)
O2—Ba1—O2^iii^	180.00 (7)		

Symmetry codes: (i) −*x*, −*y*+1, −*z*; (ii) *x*, *y*−1, *z*; (iii) −*x*, −*y*, −*z*; (iv) −*x*+1, −*y*+1, −*z*; (v) *x*−1, *y*−1, *z*; (vi) −*x*+*y*, −*x*, *z*; (vii) −*y*, *x*−*y*, *z*; (viii) *x*−*y*, *x*, −*z*; (ix) *y*, −*x*+*y*, −*z*; (x) *y*−1/3, −*x*+*y*−2/3, −*z*+1/3; (xi) *x*−*y*+2/3, *x*+1/3, −*z*+1/3; (xii) −*x*−1/3, −*y*+1/3, −*z*+1/3; (xiii) −*x*+*y*, −*x*+1, *z*; (xiv) −*y*+1, *x*−*y*+1, *z*.
